# Host-Level Susceptibility and IRF1 Expression Influence the Ability of IFN-γ to Inhibit KSHV Infection in B Lymphocytes

**DOI:** 10.3390/v14102295

**Published:** 2022-10-19

**Authors:** Nedaa Alomari, Jennifer Totonchy

**Affiliations:** Biomedical and Pharmaceutical Sciences, Chapman University, Irvine, CA 92618, USA

**Keywords:** KSHV, HHV-8, B lymphocyte, type II interferon signaling

## Abstract

Kaposi’s sarcoma-associated herpesvirus (KSHV) is associated with vascular endothelial cell tumor, Kaposi’s sarcoma (KS) and lymphoproliferative disorder, multicentric Castleman’s disease (MCD), primary effusion lymphoma (PEL) and KSHV inflammatory cytokine syndrome (KICS). Dysregulation of proinflammatory cytokines is found in most KSHV associated diseases. However, little is known about the role of host microenvironment in the regulation of KSHV establishment in B cells. In the present study, we demonstrated that IFN-γ has a strong inhibitory effect on KSHV infection but only in a subset of tonsil-derived lymphocyte samples that are intrinsically more susceptible to infection, contain higher proportions of naïve B cells, and display increased levels of IRF1 and STAT1-pY701. The effect of IFN-γ in responsive samples was associated with increased frequencies of germinal center B cells (GCB) and decreased infection of plasma cells, suggesting that IFN-γ-mediated modulation of viral dynamics in GC can inhibit the establishment of KSHV infection.

## 1. Introduction

The human gamma-herpesvirus KSHV (HHV-8) is the etiologic agent of multiple human malignancies including Kaposi sarcoma (KS) [1], primary effusion lymphoma (PEL), multicentric Castleman’s disease (MCD) and KSHV-associated inflammatory cytokine syndrome (KICS) [2,3,4]. Similar to other herpesviruses, KSHV can display latent and lytic modes of infection. KSHV exhibits complex interactions with host cells and the inflammatory microenvironment including manipulation of host cytokine production and production of viral homologs of cellular cytokines and cytokine-regulating proteins [5].

Type II interferon gamma (IFN-γ) is a pleiotropic cytokine that has antiviral activity and immunomodulatory functions. Functionally, IFN-γ can establish strong antiviral effects on virus infected cells and neighboring cells [6,7]. It can also activate and recruit immune cells, including macrophages, NK cells and dendritic cells. In addition, IFN-γ can maintain antiviral activity through promoting the differentiation and maturation of T cells and B cells [8,9,10].

Interestingly, existing data exploring the responses of KSHV-associated disorders to IFN-γ signaling has shown conflicting results with some studies showing stimulatory and inhibitory effects. One study demonstrated that recombinant IFN-γ induces KSHV lytic replication in BCBL-1 cells cocultured with HIV-1-infected T cells [11]. In contrast, IFN-γ treatment reduced the amount of KSHV infectious virus and decreased the expression of lytic-associated viral mRNAs in infected primary human lymphatic endothelial cells (LECs) as well as induced KSHV-producer cells (iSLK.219) [12]. IFN-γ also induced expression of antiviral proteins such as double-stranded RNA-activated protein kinase (PKR) [13]. These disparate responses to IFN-γ could be explained by cell-type-specific differences or different concentrations and treatment durations. Therefore, despite studies exploring the effect of IFN-γ in vitro and in vivo, it is still unclear whether IFN-γ can modulate the early establishment of KSHV infection in B lymphocytes.

In the current study, we evaluate how IFN-γ affects KSHV infection of primary B lymphocytes using our well-established model of ex vivo infection in human tonsil lymphocytes [14,15,16]. We demonstrate that IFN-γ can inhibit KSHV infection in B cells at an early stage. Interestingly, we observed that some tonsil-derived lymphocyte specimens responded to IFN-γ while others did not, and IFN-γ responsive specimens were more likely to be highly susceptible to KSHV infection. We also show that the inhibitory activity of IFN-γ is associated with an increased frequencies of germinal center B cells (GCB) and decreased infection of plasma cells. Moreover, we found that specimens enriched in immature B cell subsets with elevated expression of human IRF-1 were more likely to be IFN-γ responsive. These results identify IRF-1 expression as a novel susceptibility factor for KSHV infection in B cells, and suggest that, in highly permissive microenvironments, IFN-γ signaling in germinal centers can influence the initial course of KSHV infection in B lymphocytes.

## 2. Materials and Methods

### 2.1. Reagents and Cell Lines

CDw32 L cells (CRL-10680) were obtained from ATCC. Cells were cultured in DMEM supplemented with 20% FBS (Sigma Aldrich) and Penicililin/Streptomycin/L-glutamine (PSG/Corning). To prepare CDw32 L cells, we first trypsinized and resuspended cells in 15 mL of media in a petri dish and irradiated with 45 Gy of X-ray radiation using a Rad-Source (RS200) irradiator. Irradiated cells were then counted and cyropreserved until needed for experiments. Cell-free KSHV.219 virus derived from iSLK cells [17] was a gift from Javier G. Ogembo (City of Hope, Duarte, CA, USA). Human tonsil specimens were obtained from the National Disease Research Interchange (NDRI; ndriresource.org) or Cooperative Human Tissue Network (CHTN; www.chtn.org). Human fibroblasts were used for viral titering. Cells were derived from primary human tonsil tissue and immortalized using HPV E6/E7 lentivirus derived from PA317 LXSN 16E6E7 cells (ATCC CRL-2203). Antibodies for flow cytometry were from BD Biosciences and Biolegend and are detailed below. Recombinant Human IFN-γ protein was from Novus Biologicals.

### 2.2. Purification of Cell-Free KSHV Virions

iSLK.219 (doxycycline-inducible SLK cells harboring latent rKSHV.219) were maintained in Dulbecco’s Modified Eagle Medium (DMEM), in the presence of 10% FBS, 1% penicillin and streptomycin, G418 (250 μg/mL), hygromycin (400 μg/mL), and puromycin (10 μg/mL). For virus stock preparations, cultures at 80–90% confluence were stimulated with 3 mM sodium butyrate and 2 µM doxycycline hyclate for 72 h. At 3 days post induction, supernatants were collected and clarified by centrifugation at 1700 rpm for 12 min at 4 °C and filtered with a 0.45 µm vacuum filter. Virions were pelleted out of clarified supernatant over 25% sucrose in TNE (50 mM Tris (pH 7.4), 100 mM NaCl, 0.1 mM EDTA, pH 7.4) by centrifugation at 22,000 rpm for 2 h. Virus pellets were resuspended in TNE and stored at −80 °C. Infectious titer doses were determined by infection of human fibroblasts with varying doses of the virus stock and quantified at 3 days post infection via flow cytometry for GFP+ cells. The per-cell dose sufficient to infect 20% of fibroblasts at 3 dpi (ID20) was calculated via linear regression and used to infect B lymphocytes.

### 2.3. Isolation of Primary Lymphocytes from Human Tonsils

De-identified human tonsil specimens were obtained after routine tonsillectomy by NDRI or CHTN and shipped overnight on wet ice in DMEM+PSG. All specimens were received in the laboratory less than 24 h post-surgery and were kept at 4 °C throughout the collection and transportation process. Lymphocytes were extracted by dissection and maceration of the tissue in RPMI media. Lymphocyte-containing media was passed through a 40 µm filter and pelleted at 1500 rpm for 5 min. Red blood cells (RBC) were lysed for 5 min in sterile RBC lysing solution (0.15 M ammonium chloride, 10 mM potassium bicarbonate, 0.1 M EDTA). After dilution to 50 mL with PBS, lymphocytes were counted, and pelleted. Aliquots of 5(10)7 to 1(10)8 cells were resuspended in 1 mL of freezing media containing 90% FBS and 10% DMSO and cryopreserved until needed for experiments.

### 2.4. Infection of Primary Lymphocytes with KSHV

Lymphocytes were thawed rapidly at 37 °C, diluted dropwise to 5 mL with RPMI and pelleted. Pellets were resuspended in 1 mL RPMI + 20% FBS + 100 µg/mL DNaseI+ 100 µg/mL Primocin (Invivogen) and allowed to recover in a low-binding 24 well plate for 2 h at 37 °C, 5% CO_2_. After recovery, total lymphocytes were counted and naïve B cells were isolated using the Stemcell Robosep magnetic cell separation (Cat#21000) according to manufacturer instructions. Bound cells (non-naïve B and other lymphocytes) were retained and kept at 37 °C in RPMI + 20% FBS + 100 µg/mL Primocin during the initial infection process. 1(10)^6^ isolated naïve B cells were infected with iSLK-derived KSHV.219 (per cell dose equivalent to the ID20 at 3 dpi on human fibroblasts, see Section 2.2) or Mock infected in a total of 400 µL serum free RPMI in 12 mm × 75 mm round bottom tubes via spinoculation at 1000 rpm for 30 min at 4 °C followed by incubation at 37 °C for an additional 30 min. Following infection, cells were plated on irradiated CDW32 feeder cells in a 48 well plate, reserved bound cell fractions were added back to the infected cell cultures, and FBS and Primocin were added to final concentrations of 20% and 100 µg/mL, respectively and recombinant cytokines were also added at this stage, depending upon the specific experiment. Cultures were incubated at 37 °C, 5% CO_2_ for the duration of the experiment. At 3 days post-infection, cells were harvested for analysis by flow cytometry and supernatants were harvested, clarified by centrifugation for 15 min at 15,000 rpm to remove cellular debris, and stored at −80 °C for analysis.

### 2.5. Flow Cytometry Analysis

Approximately 5 × 10^5^ lymphocytes were aliquoted into a 96-well round bottom plate and at day 0 (baseline) or at 3 days post-infection (3 dpi), pelleted at 1500 rpm for 5 min. Pellet was resuspended with 100 µL PBS containing zombie violet fixable viability stain (Biolegend Cat# 423113) followed by incubation on ice for 15 min. After incubation, cells were pelleted at 1500 rpm for 5 min and resuspended in 100 µL PBS, containing the following: 2% FBS, 0.5% BSA and 0.1% sodium azide (FACS Block) and followed by incubation on ice for 10 min. Cells were pelleted at 1500rpm for 5 min and resuspended in 50 μL of PBS with 0.5% BSA and 0.1% sodium azide (FACS Wash), containing antibody cocktails, 10 µL BD Brilliant Stain Buffer Plus (BD 566385) and antibodies as follows: IgD-BUV395 (2.5 µL/test BD 563823), CD77-BV510 (2.0 µL/test BD 563630), CD138-BV650 (2 µL/test BD 555462), CD27-BV750 (2 µL/test BD 563328), CD19-PerCPCy5.5 (2.0 µL/test BD 561295), CD38-APC (10 µL/test BD 560158), CD20-APCH7 (2 µL/test BL 302313). For IRF-1 baseline and STAT1-pY701 samples were stained and analyzed as above with phenotype antibody panel as follows: 510 fixable viability stain, IgD-BUV395 (2.5 µL/test BD 563823), CD138-BUV737 (2 µL/test BD 612834), CD27-BV750 (2 µL/test BD 563328), CD19-PerCPCy5.5 (2.0 µL/test BD 561295), CD38-Alexa700 (2.0 µL/test Biolegend 356624) cells were fixed for 10 min in BD cytofix/cytoperm (51-2090KZ), pelleted and further treated for 10 min with cytofix/cytoperm+10% DMSO (super perm). Intracellular antibodies, as follows, were diluted in 1x BD Permwash (51-2091KZ) and left on fixed cells overnight at 4 °C. IRF-1-PE (2.0 µL/test BD 566322), BV421 STAT1-pY701 (2.0 µL/test BD 566238). After incubation, 150 µL FACS Wash was added. Cells were pelleted at 1500 rpm for 5 min followed by two washes using FACS Wash. Cells were collected in 200 µL FACS Wash for flow cytometry analysis. Cells and appropriate compensation controls were acquired using an LSR Fortessa X-20 cell analyzer (BD Biosciences). Acquisition settings were applied to allow recording of all events present in the sample, so absolute numbers of events acquired varies between samples but is never less than 50,000 events. BD CompBeads (51-90-9001229) were used to calculate compensation for all antibody stains and methanol-fixed Namalwa cells (ATCC CRL1432) +/− KSHV were used to calculate compensation for GFP and the fixable viability stain. Flow cytometry data was analyzed using FlowJo software and exported for analysis in R Software. as described below.

### 2.6. Statistical Analysis

Statistical analysis and plots were performed in Rstudio software using ggplot2 [18], ggcorrplot [19], ggally [20], tidyverse [21] and rstatix packages [22]. Specific methods of statistical analysis including Anova, Wilcoxon rank sum test, Pearson and Spearman correlation are detailed in the figure legands.

## 3. Results

### 3.1. IFN-γ Inhibits KSHV Infection in Tonsil-Derived Lymphocytes That Are Highly Susceptible to Infection

**Figure 1:**
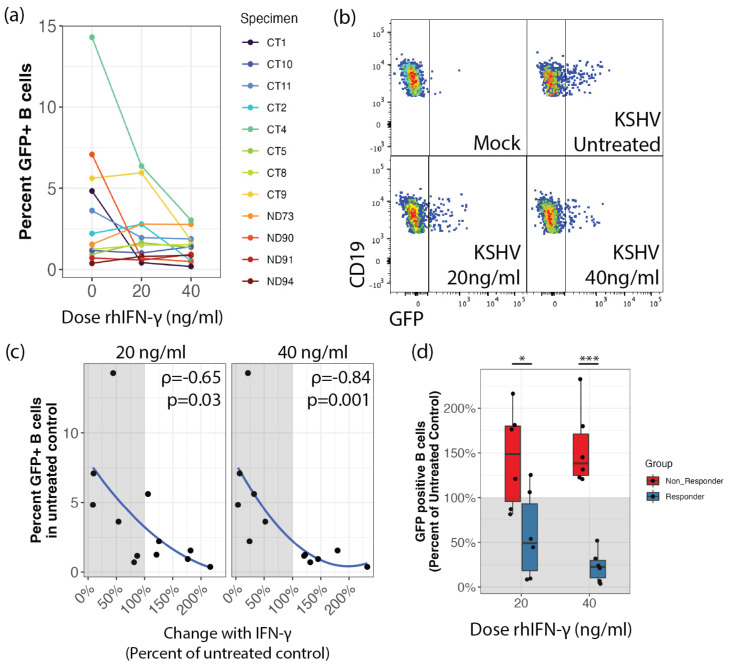
IFN-γ inhibition of KSHV infection in B cells is restricted to susceptible tonsil-derived lymphocyte specimens. B cells derived from 12 unique tonsil specimens were infected with KSHV and cultured in a reconstituted total lymphocyte environment with the indicated doses of IFN-γ. At 3dpi cells were analyzed for KSHV infection by flow cytometry. (**a**) the percentage of GFP+ cells within the viable CD19+ B cell population. (**b**) representative flow cytometry data showing decreased viable/CD19+/GFP+ cells in response to IFN-γ treatment (**c**) monotonic relationship between GFP+ cells in untreated control (specimen susceptibility) and the per-specimen change in GFP+ B cells with IFN-γ treatment. Spearman rank correlation coefficients and p-values for each dose are indicated on the respective panels. (**d**) analysis of grouped differences between responder/susceptible (blue) and non-responder/refractory (red) specimens at indicated doses of IFN-γ represented as a percent of untreated control with grey shading indicating decreased relative values. Wilcoxon rank sum test *p*-values * = 0.04; *** = 0.002.

We hypothesized that IFN-γ could alter the early stages of KSHV infection in B cells in tonsil-derived lymphocyte ex vivo cultures. In order to test this, we performed Mock or KSHV infections with tonsil-derived lymphocytes from 12 unique donors, and treated the resulting cultures with different concentrations of IFN-γ. At 3 dpi, lymphocytes were analyzed by flow cytometry for KSHV infection based on GFP expression and B cell immunological lineage markers shown in Table 1. These results show a reduction in KSHV infection in a subset of samples when treated with IFN-γ (Figure 1a,b). We previously demonstrated that human tonsil-derived lymphocytes display variable susceptibility to KSHV infection in our ex vivo infection model and have hypothesized that host-level factors contribute to this variable susceptibility [14]. Interestingly, in this dataset, samples that were more susceptible in the untreated conditions appeared more responsive to IFN-γ-mediated repression while samples with low infection frequencies in untreated samples did not respond to IFN-γ. Indeed, when we removed the sample-specific variability by calculating the IFN-γ response as a percent of untreated control infection for each sample, we observed statistically significant correlations between the inhibitory effect of IFN-γ and the total GFP positive B cells in the untreated conditions with a monotonic relationship between the variables, thus statistically validating the observation that susceptibility to KSHV infection is related to responsiveness to IFN-γ treatment (Figure 1c). When we divided the samples into “responder” and “non-responder” groups based on this observation and examined the inhibitory effect of IFN-γ, we observed dose-dependent decreases in infection with IFN-γ treatment in responder samples while non-responder samples showed a modest increase (above 100% of control) in KSHV infection with IFN-γ treatment (Figure 1d). The difference in IFN-γ inhibitory activity between responder/susceptible and non-responder/refractory samples was dose-dependent and statistically significant at both the 20 µg/mL (*p* = 0.04) and 40 µg/mL doses (*p* = 0.002). Taken together, these results demonstrate that IFN-γ is capable of inhibiting KSHV infection in B lymphocytes, but only in tonsil-derived lymphocyte specimens that are intrinsically susceptible to infection.

### 3.2. IFN-γ Decreases the Frequency of Infected Plasma Cells in Responsive Samples

**Figure 2:**
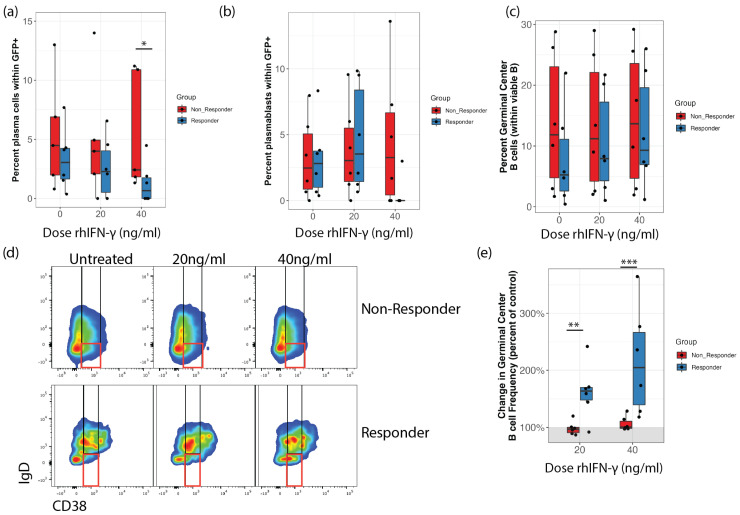
IFN-γ inhibition of KSHV infection in B cells is associated with decreased infection of plasma cells and increased germinal center B cell frequencies. B cells derived from 12 unique tonsil specimens were infected with KSHV and cultured in a reconstituted total lymphocyte environment with the indicated doses of IFN-γ. At 3dpi cells were analyzed for KSHV infection (GFP+) and B cell immunophenotypes by flow cytometry. Samples were grouped into responder (blue) and non-responder (red) based on data in Figure 1 (**a**) the percentage of plasma cells within GFP+ cells. Wilcox test * *p* = 0.05 comparing groups at 40 ng/mL (**b**) the percentage of plasmablasts within GFP+ cells. (**c**) frequency of germinal center B cells within viable B Repeated measures ANOVA significant effect of IFN-γ treatment in responders only *p* = 0.009 (**d**) representative flow cytometry data showing the frequency of germinal center B cells (red gates; see Table 1 for detailed subset definitions) within viable B in a non-responder (top) and responder (bottom) sample at indicated doses of IFN-γ. (**e**) data as in (**c**) normalized to untreated controls (IFN-γ treated/sample-matched control). Wilcox test ** *p* = 0.03, *** *p* < 0.01.

In order to begin examining potential mechanisms for the inhibitory effect of IFN-γ in susceptible samples, we examined whether IFN-γ treatment resulted in significantly different distribution of B cell subsets within the KSHV-infected population (subsets within GFP+). Two-way repeated measures ANOVA revealed a significant effect of IFN-γ treatment on the frequency of infected plasmablasts in IFN-γ responsive cultures. Unpaired wilcox test comparing the responder vs. non-responder groups showed significantly decreased representation of plasma cells within the infected cell population in responders vs. non-responders at the 40ng/mL dose (Figure 2a). Infected plasmablasts were also decreased, with only one sample in the responder group showing any detectable GFP+ plasmablasts at the 40ng/mL dose, but the difference between the groups was not statistically significant (Figure 2b).

### 3.3. IFN-γ Increases Germinal Center B Cell Frequencies in Responsive Samples

We then examined whether responsive samples had significantly altered B cell subset frequencies in response to IFN-γ stimulation. Interestingly, in untreated, KSHV infected cultures, we observed decreased germinal center B cell (GCB) frequencies in IFN-γ responsive samples compared to non-responders and IFN-γ treatment eliminated this difference (Figure 2c,d). Indeed, there was a significant effect of IFN-γ on GCB frequency via repeated measures ANOVA in the responder samples only (*p* = 0.009). When we plotted the effect of IFN-γ treatment on GCB frequencies as a percent of the untreated control, there is a significant difference between the two groups of specimens, with no effect in non-responders and significant increases in GCB in responder samples at both IFN-γ doses (Figure 2e).

### 3.4. Response to IFN-γ Stimulation Is Associated with Samples That Have Increased Frequency of Immature B Cells and High Baseline IRF1 Expression

**Figure 3:**
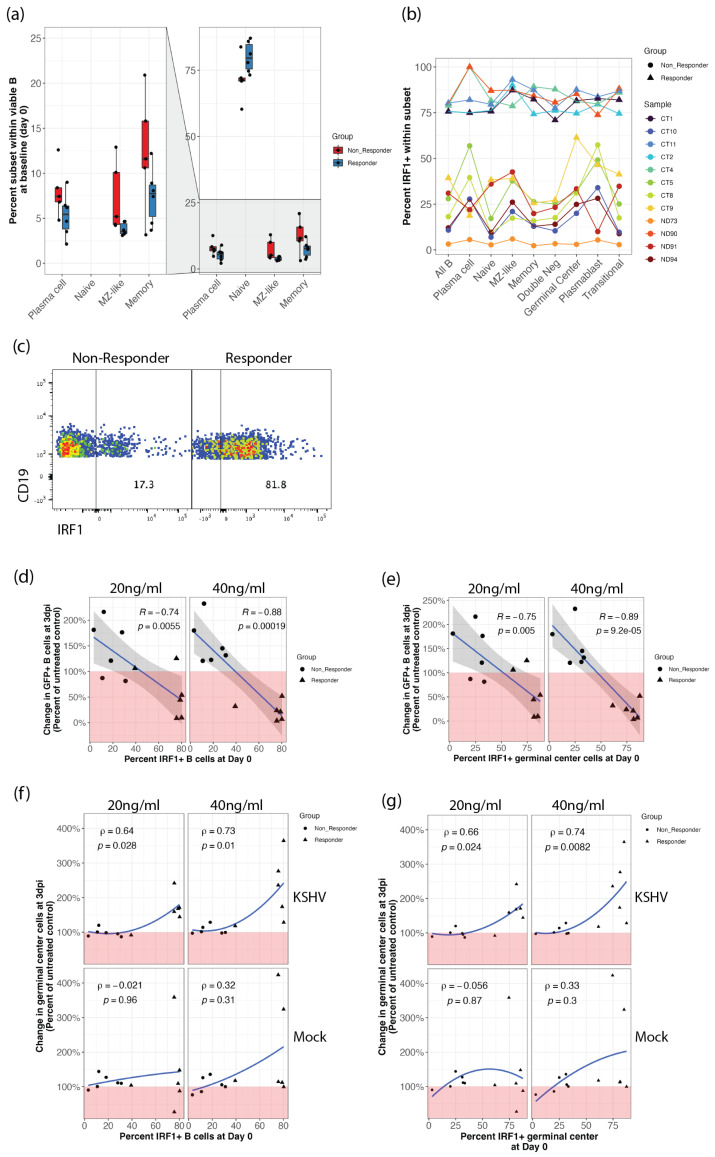
IFN-γ responsive specimens have higher baseline levels of immature and IRF1+ B cells. Tonsil-derived lymphocytes used in infection experiments in Figure 1 and Figure 2 were analyzed by flow cytometry at day 0 for B cell immunophenotypes and IRF1 expression. (**a**) distribution of B cell subset frequencies within viable CD19+ comparing responder (blue) and non-responder (red) groups Wilcoxon test *p* < 0.01 and effect size > 0.5 for all subsets shown (**b**) percent IRF1+ cells within B cell subsets (*x*-axis) for each sample (indicated by point and line color) classified based on IFN-γ responders (triangles) and non-responders (circles) shown in Figure 1c. Wilcoxon test comparing responders to non-responders *p* << 0.001 and effect size > 0.6 for all B cell subsets. (**c**) representative flow cytometry data showing baseline IRF1 expression within viable, CD19+ cells from a non-responder and responder samples. Pearson correlations between the effect of IFN-γ doses on KSHV infection at 3 dpi and (**d**) IRF1 expression in all B cells or (**e**) IRF1 expression in GCB at day 0. Spearman correlations between the effect of IFN-γ doses on GCB frequency in mock infected (bottom) and KSHV-infected (top) cultures at 3 dpi and (**f**) IRF1 expression in all B cells or (**g**) IRF1 expression in GCB at day 0.

We next performed B cell immunophenotyping at day 0 to examine whether susceptible, IFN-γ responsive samples had distinctive baseline frequencies of B cell subsets compared to non-responders. We observed that IFN-γ responders displayed significantly higher frequencies of naïve B cells and lower frequencies of mature subsets including MZ-like, memory and plasma cells (Figure 3a), indicating that the IFN-γ responsive phenotype may be mediated by signaling to immature B cell subsets.

IRF-1 expression is necessary to mediate IFN-γ antiviral effects in primary macrophages replicating MNV [23], WNV [24] and MHV68 [25]. To assess whether IRF-1 facilitates the antiviral effects of IFN-γ, we examined the subset-specific expression of IRF-1 in human tonsil-derived B cells. Interestingly, this sample cohort contained two distinct groups based on IRF1 expression at baseline (day 0) with one group having IRF1 expression uniformly above 60% in all B cell subsets and the other group with IRF1 expression below 60%, Importantly, all IRF1 high samples were IFN-γ responders while, with one exception, the IRF1 low samples were non-responders (Figure 3b,c). A Wilcoxon one sample signed rank test revealed statistically significant differences between responders and non-responders for IRF1 expression at baseline (*p* << 0.001 and effect size > 0.6 for all B cell subsets). We next examined whether IRF1 expression was correlated with increased GCB frequency (Figure 2c–e) and decreased overall infection (Figure 1d) that we observed for the IFN-γ response in KSHV infected cultures. This analysis revealed significant linear correlations between the change in total infection at 3dpi with IFN-γ treatment and both total IRF1+ B cells (Figure 3d) and IRF1+ GCB (Figure 3e) at day 0 with more robust correlations observed at higher concentrations of IFN-γ, mirroring the dose responses we observed in Figure 1d. IFN-γ-mediated increases in total GCB frequencies were also correlated with both total IRF1+ B cells (Figure 3f) and IRF1+ GCB (Figure 3g) at day 0. These relationships were similarly dose-dependent but more monotonic than linear. Interestingly, the same relationships between IRF1 and GCB frequencies were not present in IFN-γ treated mock infected cultures (Figure 3f,g, bottom panels), suggesting that IFN-γ is inhibiting a virus-specific effect on GCB frequencies in susceptible samples.

### 3.5. STAT1 Phosphorylation Is Elevated in IFN-γ Responsive Samples and KSHV Infection Increases STAT1 Phosphorylation Response to IFN-γ Treatment in GCB

**Figure 4:**
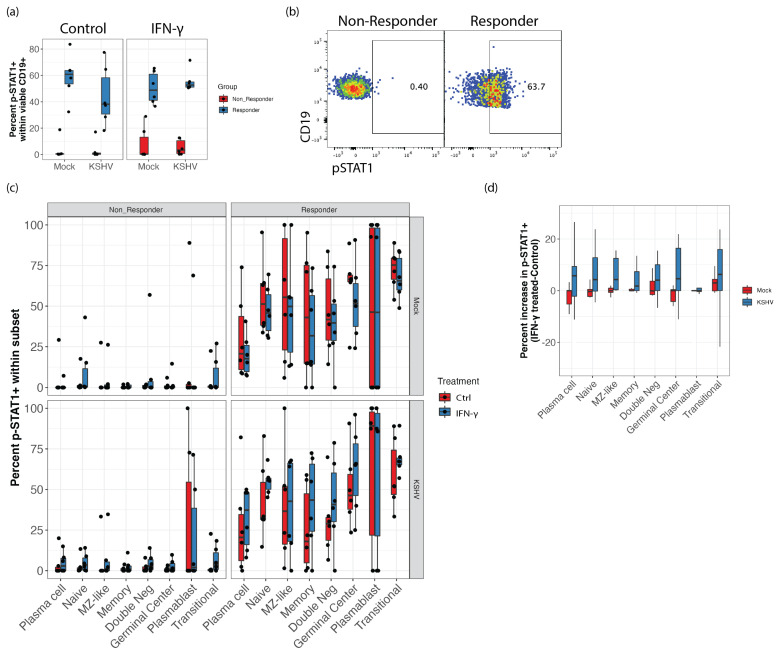
IFN-γ responsive samples have higher baseline pSTAT1. Phospho-flow cytometry for STAT1 Y701 (**a**) total pSTAT1 in responder (blue) vs. non-responder (red) groups under different culture conditions. Unpaired Wilcoxon test *p* = 0.002 comparing responder to non-responder groups for all conditions shown (**b**) representative flow cytometry plots showing pSTAT1 staining in untreated Mock samples for a responder and non-responder sample (**c**) pSTAT1 in B cell subsets in different culture conditions (facets) with (blue) or without (red) IFN-γ treatment. Unpaired Wilcoxon test comparing responder and non-responder groups were statistically significant (*p* > 0.01, effect size > 0.6) for all comparisons except plasmablasts (**d**) Increase in pSTAT1 with IFN-γ treatment (treated-control) for B cell subsets comparing mock and KSHV-infected cultures (color) in responder group samples only. Comparisons in (**d**) are not statistically significant.

IRF1 and STAT1 function cooperatively to potentiate IFN-γ responses [26]. In order to see whether STAT1 was involved in mediating the response of KSHV infection to IFN-γ, we performed phospho-flow cytometry to examine phosphorylation of STAT1 on Y701 (pSTAT1) in Mock and KSHV infected cultures with and without IFN-γ treatment at 40ng/mL. Because pSTAT1 in response to IFN-γ treatment is likely transient, we analyzed these experiments at 24 h post-infection. Interestingly, we observed that IFN-γ responsive samples had higher pSTAT1 compared to non-responder specimens even in the absence of IFN-γ treatment or KSHV infection (Figure 4a). When we examined the distribution of pSTAT1 within B cell subsets in these cultures, non-responder specimens had uniformly lower levels of pSTAT1 in untreated cultures and were poorly responsive to IFN-γ stimulation (Figure 4b, left panels). Comparing each subset between the non-responders (Figure 4b, left panels) and the responders (Figure 4b, right panels) revealed that nearly every subset had significantly higher pSTAT1 in responder samples regardless of IFN-γ treatment or KSHV infection (*p* < 0.03, effect size > 0.45). Only plasmablasts in KSHV infected cultures showed similar pSTAT1 comparing non-responder and responder groups (Figure 4b, bottom panels). Moreover, within the IFN-γ responsive group, KSHV-infected cultures generally showed greater increases in pSTAT1 with IFN-γ treatment (Figure 4c), indicating that infection increases the responsiveness of B cells to IFN-γ in susceptible samples. However, there was no statistically significant effect of IFN-γ treatment in these data, indicating that STAT1 signaling maybe only a minor contributor to the IFN-γ mediated effect on KSHV infection.

## 4. Discussion

KSHV has been known to infect several cell types including human B cells [27]. KSHV is able to downregulate antiviral immunity and evade immune surveillance, allowing it to establish a life-long infection. Several findings illustrate that KSHV alters cytokine production and KSHV-associated diseases are often accompanied by altered inflammatory states as a result of cytokine dysregulation [28,29]. However, how host inflammatory mediators influence susceptibility to KSHV infection and the initial stages of infection remains undefined. IFN-γ signalling is a well-established innate antiviral mechanism, and in this study we show that, in certain contexts, IFN-γ can also inhibit KSHV infection in B lymphocytes derived from human tonsil. Moreover, the results presented in this study are the first to identify IRF1 as a factor associated with KSHV-susceptible samples that can influence the inhibitory activity of IFN-γ in the context of early infection of B cells.

The majority of studies exploring the effect of IFN-γ on gamma-herpesvirus biology have been in the context of an established infection. IFN-γ treatment inhibited KSHV replication in primary human LECs and KSHV-producer cell line (iSLK.219) [12]. MHV-68 uses IFN-γ responsive promoter elements to repress expression of the lytic switch protein ORF50 [30]. One study showed that co-treatment of MHV-68 infected mice with IL-4 and IFN-γ inhibitors is necessary to reactivate the virus [31] while other studies have shown that IFN-γ reactivates MHV-68 in macrophages but not B cells [30,32]. It was also shown that IFN-γ-mediated inhibition of MHV-68 reactivation in macrophages can be controlled by toll-like receptor activation, suggesting that the activation state of infected cells can control responsiveness to IFN-γ signals [33].

The focus of our laboratory’s research has been evaluating early events in KSHV infection in human tonsil-derived lymphocytes and understanding how host factors contribute to these early dynamics to either promote or inhibit the establishment of KSHV infection. We have previously shown that KSHV establishes infection in CD138 plasma cells at early timepoints [14]. We also recently showed that supplementation of lymphocyte cultures with IL-21 promotes the establishment of infection and that this effect is associated with IL-21 mediated differentiation of naïve B cells into plamsa cells [15]. Thus, our previous studies are building a model in which the early differentiation of B cells influences the magnitude of KSHV infection. This is similar to the well-characterized mechanisms in MHV-68 and EBV where infected cells transit the germinal center to establish latent infection in memory B cells or lytic infection in plasma cells for dissemination [34,35]. In this study, we show that IFN-γ-mediated inhibition of early infection events is associated with increased GCB frequencies and decreased infection in plasma cells (Figure 2). This result may suggest that IFN-γ inhibits or alters differentiation of KSHV-infected cells at the GCB stage and limits efficient establishment by preventing the emergence of infected plasma cells at early timepoints.

IRF-1 repression was recently shown to exacerbate and extend MHV-68-mediated germinal center expansion during the early stages of infection, and decreased IRF1 expression is associated with gamma-herpesvirus associated posttransplant lymphoproliferative disorder in humans [36]. In the current study IRF1 was highly expressed in KSHV-susceptible samples that had low GCB frequencies and were responsive to inhibition by IFN-γ while refractory, non-responsive samples had lower expression of IRF1 and higher frequencies of GCB in unstimulated controls. In the context of the current literature, these observations imply that geminal center dynamics can substantially influence the early stages of KSHV infection in human lymphocytes.

Our data showed both elevated STAT1 phosphorylation and elevated IRF1 in IFN-γ responsive samples. Previous studies have shown that IRF1-regulated gene products can increase STAT1 phosphorylation and DNA binding in HEK293 cells [37]. However, we do not observe a direct correlation between levels of IRF1 and STAT1 in our data, suggesting that increased pSTAT1 is not a direct result of increased IRF1 expression in responding tonsil-derived lymphocytes.

Additional study is needed to understand these mechanisms in more detail. Interestingly, studies have demonstrated cross-talk between IL-21, IFN-γ and IL-4 in germinal center B cells in the context of TLR stimulation, and the generation Tbet+ or Tbet+/CD11c+ memory B cells is governed by this cytokine milieu [38]. Tbet+ memory B cells driven by IFN-γ have recently been implicated in antiviral responses [38]. as well as autoimmunity [39]. Tbet+/CD11c+ B cells generated in the presence of IL-21 and the absence of IL-4 are referred to as age-associated B cells, and are implicated in immunosenescence in addition to being elevated in autoimmune and autoinflammatory diseases [40]. Our previous results [15] and those presented in this study may indicate that KSHV exploits these differentiation pathways during the establishment of infection in a new human host, creating a population of infected B cells that are poised to promote autoimmune, pro-inflammatory and lymphoproliferative states in the context of immune dysfunction.

## Figures and Tables

**Table 1:** Lineage definitions for lymphocyte subsets used in the study.

B Lymphocytes	
Subset	Molecular Markers
Plasma	CD19^+^, CD20^+/−^, CD138^+(**Mid to High**)^, CD38^−^
Transitional	CD19^+^, CD138^−^, CD38**^Mid^**, IgD^+ (**Mid to High**)^
Plasmablast	CD19^+^, CD138^−^, CD38**^High^**, IgD^+/− (**mostly**−)^
Germinal Center	CD19^+^, CD138^−^, CD38**^Mid^**, IgD^−^
Naïve	CD19^+^, CD138^−^, CD38**^Low^**, CD27^−^, IgD^+ (**Mid to High**)^
Marginal Zone Like (MZ-Like)	CD19^+^, CD138^−^, CD38**^Low^**, CD27^+ (**Mid to High**)^, IgD^+ (**Mid to High**)^
Memory	CD19^+^, CD138^−^, CD38**^Low^**, CD27^+ (**Mid to High**)^, IgD^−^
Double Negative	CD19^+^, CD138^−^, CD38**^Low^**, CD27^−^, IgD^−^

**Note:** Subset definitions for B cell subsets used in this study.

## Data Availability

Raw data files collected for this study are available upon request from the corresponding author without restriction.
